# A Case of Tumor of Follicular Infundibulum Involving the Vulva

**DOI:** 10.1155/2019/4606493

**Published:** 2019-01-28

**Authors:** Victor Santiago, David Cartwright, Pooria Khoshnoodi, Molly Klein, Alessio Giubellino

**Affiliations:** Departments of Laboratory Medicine and Pathology, University of Minnesota, Minneapolis, MN, USA

## Abstract

Tumor of the follicular infundibulum or infundibuloma is a relatively rare benign adnexal tumor usually solitary and located in the head, neck, and trunk. Here we present a 70-year-old woman with a tender vulvar lesion. Histopathologic exam shows a well-circumscribed lesion with a subepidermal horizontally oriented, plate-like proliferation of pale appearing squamous epithelial cells with numerous points of connections with the overlying epidermis and peripheral palisading. Overall these histopathologic features are consistent with the diagnosis of tumor of follicular infundibulum involving genital skin.

## 1. Introduction

Tumor of the follicular infundibulum (TFI), also known as infundibuloma, is a rare benign neoplasm described in 1961 by Mehregan and Butler [1] with a recognized follicular isthmic differentiation, specifically from the outer root sheath. Hence, the lesion would be better identified as isthmicoma, although this term is not commonly used. The lesion, which can be solitary or multiple [2, 3], can have variable clinical presentations. The most common form of the tumor is an asymptomatic solitary hypopigmented macule, plaque, papule, or nodule, sometimes appearing like scar or atrophic and usually less of 1 cm in greatest dimension. Pigmented lesions have been also described [4] but appear to be less common. The lesion can also be scaly. It affects women in midlife or elderly more than men and usually is located on the head, neck, and trunk areas. While multiple lesions are usually sporadic [5], an association has been reported with Cowden syndrome or arising within a nevus sebaceous [6]. It is also one of the epithelial tumors arising in the Schopf-Schulz-Passarge syndrome [7]. Rare cases with eccrine or sebaceous differentiation have been described [8, 9].

The variable clinical presentation is usually resolved by a biopsy with evaluation of distinct histopathological features. Crucial histologic findings include a distinctive outline with epidermal-bound dermal horizontal proliferation of squamous epithelial cells with small monomorphic nuclei and abundant eosinophilic pale cytoplasm. Interconnecting bands of epithelial cells give the lesion a fenestrated or reticulated pattern appearance. Numerous keratocysts are present at the base of the lesion and a patchy eosinophilic basement membrane is notable around tumor islands. To our knowledge, this is the first solitary infundibuloma reported in genital skin.

## 2. Case Report

A 70-year-old woman with a history of diabetes mellitus type 2, hypertension, gastroesophageal reflux disease, and hypothyroidism presented with urinary incontinence. Evaluation showed a tender left vulvar lesion, which she stated had worsened over the prior nine months. The clinical impression of the vulvar lesion was lichen sclerosus et atrophicus. The patient was using an over-the-counter topical treatment (zinc oxide cream), with no amelioration of symptoms. Later, triamcinolone acetonide was tried, without improvement.

**Figure 1:**
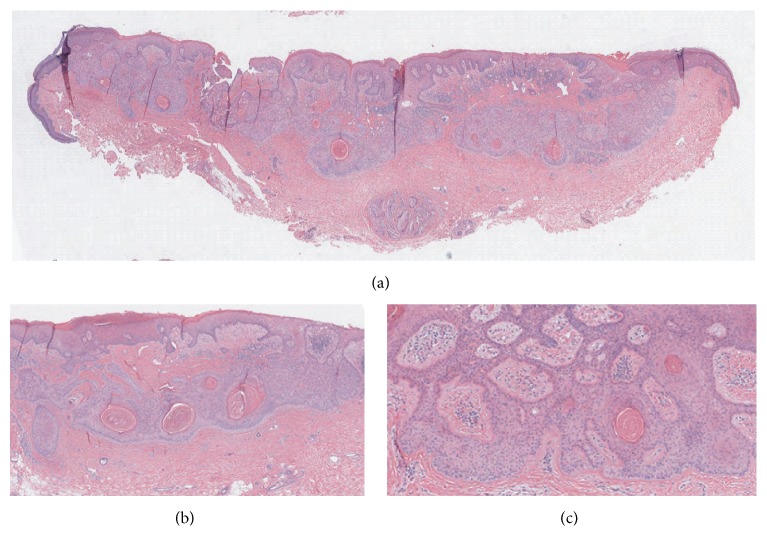
Low power (2x) view of the entire biopsy demonstrating a classic plate-like proliferation of keratinocytes parallel to the epidermis (a). Higher power (10x) of another section of the tumor shows the presence of numerous keratocysts (b) and a net-like architecture with frequent connections of the plate to the above epidermis (c).

**Figure 2:**
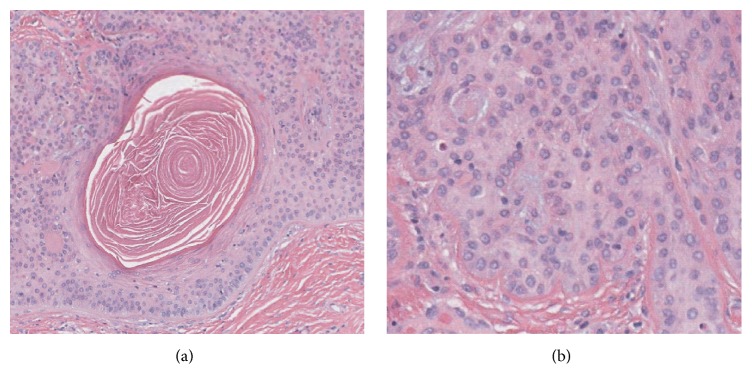
Higher power (20x) shows a keratocyst with no visible granular layer and abrupt keratinization (a). The pale pink cytoplasm of tumor cells is evidence of tumor glycogenation and the characteristic differentiation (b).

Over a period of months, the lesion slightly decreased in size but irritation and tenderness increased. Physical examination before the biopsy showed a one-centimeter ulcerated lesion with lichenoid change, involving introitus and clitoral hood. A biopsy was performed and histopathologic examination showed an overall well circumscribed lesion (Figure 1) with a subepidermal, horizontally oriented, plate-like proliferation of pale appearing squamous epithelial cells, with numerous points of connection with the overlying epidermis and peripheral palisading (Figure 1). Interconnecting bands of epithelial cells give the lesion a fenestrated or reticulated pattern appearance. Numerous keratocysts are noted at the base, while, notably, no visible granular layer is present (Figure 2). An eosinophilic basement membrane is notable around tumor islands. Elastic fibers appear to be condensed below the lesion and a patchy and mild lymphocytic infiltrate is also present around and within the tumor.

## 3. Discussion

Vulvar skin biopsies are a relatively common source of intradepartmental consultation to dermatopathologists, ranging from inflammatory lesions to neoplastic proliferations, including benign adnexal tumors. Here we describe the occurrence of a tumor of follicular infundibulum in the vulva, an unusual location for this rare benign adnexal tumor with very distinct histopathology. The crucial histopathologic changes are the formation of a plate-like proliferation of pale-appearing squamous cells, interconnected with the overlying epidermis at multiple points. Peripheral palisading can be present and the basement membrane is frequently thick. The pink and pale appearance of the keratinocytes is due to the presence of glycogen and the differentiation of these cells towards the outer root sheath at the isthmus level. In fact, occasionally this tumor is connected to follicular external root sheath of nearby vellus hairs. PAS may be used to highlight the intracytoplasmic glycogen.

The pathogenesis of this tumor is controversial, but some authors have suggested that TFI may be an epidermal reaction pattern to dermal fibrosis [10]. Although the general architectural and histopathologic pattern point to a diagnosis of infundibuloma, we also considered the possibility of a collision tumor. Indeed, the presence of numerous keratocysts at the bottom of the lesion raises the possibility of a collision with a trichoadenoma which is a tumor with a differentiation towards the infundibular portion of the hair follicle. However, no granular layer is seen (Figure 2(a)) as you expect to see in keratocyst of trichoadenoma and an abrupt type of keratinization is seen.

The histopathologic differential diagnosis may include also a superficial type of basal cell carcinoma; indeed, Weyers et al. [11] think that TFI belongs to the basal cell carcinoma (BCC) spectrum and may develop to a more aggressive type of BCC. Also, fibroepithelioma of Pinkus may mimic the fenestrated pattern of TFI, but the latter does not have the characteristic budding of basaloid cells along the branches of the interconnected epithelial strands (a sign of the germinative differentiation of the former entity), although they both have fibrotic stroma. Basaloid follicular hamartoma can also be a consideration. A lesion described as basal cell hamartoma with follicular differentiation [12] may actually represent a variant of infundibuloma with presence of structures resembling hair follicle papillae, as described also in a case of typical solitary TFI [6]. Finally, histologically this lesion may be distinguished from an adenoid type of seborrheic keratosis; the reticulated pattern of interconnecting epithelial stands and the presence of horn pseudocysts can help in this differential diagnosis.

Clinically, the differential diagnosis for this rare tumor in this location may include lichen planus, lichen sclerosus, lichen simplex chronicus, and pemphigus. Treatment for this benign lesion is surgical excision, which it is curative.

This tumor is quite underreported in the literature, likely because of its rarity and its benign nature. While prior multiple lesions have been reported [13], to our knowledge, this is the first report of an isolated single infundibuloma in the vulva and, in general, in genital skin. Although quite rare, it should be considered in the differential diagnosis when characteristic histopathologic findings are present, even when the site is unusual.
